# Efficacy of a New Alcohol-Free Organic Acid-Based Hand Sanitizer against Foodborne Pathogens

**DOI:** 10.3390/toxics11110938

**Published:** 2023-11-17

**Authors:** Gözde Bayer, Amirreza Shayganpour, Ilker S. Bayer

**Affiliations:** 1 DS Bio ve Nanoteknoloji A. Ş, Lavida City Plaza 45/7, 06530 Ankara, Türkiye; gozde.bayer@dstrace.com; 2 Smart Materials, Istituto Italiano di Tecnologia, Via Morego 30, 16163 Genova, Italy; amirreza.shayganpour@iit.it

**Keywords:** hand sanitizer, povidone-iodine, citric acid, lactic acid, azelaic acid, foodborne pathogen

## Abstract

In light of the global health crisis triggered by the COVID-19 pandemic, numerous experts have deemed the utilization of hand sanitizers imperative as a precautionary measure against the virus. Consequently, the demand for hand sanitizers has experienced a substantial surge. Since the beginning of 2020, the utilization of alcohol-free hand sanitizers has been increasingly favored due to the potential risks associated with alcohol poisoning, flammability, as well as the adverse effects on skin lipid dissolution, dehydration, and sebum reduction, which can lead to severe cases of eczema and norovirus infections. In this study, we developed an aqueous hand sanitizer that does not contain alcohol. The sanitizer consists of naturally occurring, food-grade organic acids, including lactic, citric, and azelaic acids. Additionally, food-grade ammonium sulfate and a small amount of povidone-iodine (PVPI) were included in the formulation to create a synergistic and potent antibacterial effect. The effectiveness of the hand sanitizer was evaluated against four common foodborne pathogens, namely *Clostridium botulinum*, *Escherichia coli*, *Listeria monocytogenes*, and *Staphylococcus aureus*, via in vitro testing. The organic acids exhibited a synergistic inhibitory function, resulting in a 3-log reduction in CFU/mL. Furthermore, the presence of povidone-iodine and ammonium sulfate enhanced their antibacterial effect, leading to a 4-log reduction in CFU/mL. The hand sanitizer solution remained stable even after 60 days of storage. During this period, the detection of additional triiodide (I_3_^−^) ions occurred, which have the ability to release broad-spectrum molecular iodine upon penetrating the cell walls. This alcohol-free hand sanitizer may offer extended protection and is anticipated to be gentle on the skin. This is attributed to the presence of citric and lactic acids, which possess cosmetic properties that soften and smoothen the skin, along with antioxidant properties.

## 1. Introduction

Foodborne illnesses typically manifest as either infectious or toxic in nature, arising from the infiltration of bacteria, viruses, parasites, or chemical substances into the human body via contaminated food [1,2,3,4,5]. The presence of chemical contaminants can result in acute poisoning or the development of chronic ailments, including cancer [6]. Numerous foodborne diseases have the potential to inflict enduring disabilities and even mortality [7,8,9]. Hand hygiene, specifically using hand washing, is widely regarded as the most crucial practice for preventing the transmission of pathogens [10,11,12,13]. The “gold standard” method for removing dirt and transient microorganisms from hands is via hand washing with water and soap. While plain soaps possess minimal or no antimicrobial activity against bacteria and viruses, their surfactant action, coupled with friction and final rinsing under water, can effectively eliminate dirt, soil, and microbes from the outer layer of hand skin [14,15,16,17]. In recent years, there has been a growing interest in the use of hand cleansing products with antimicrobial properties, such as antimicrobial soaps or instant hand sanitizers, which include both alcohol-based and alcohol-free preparations [18,19,20,21,22,23,24].

Alcohol-based hand sanitizers are considered to be immediate solutions for maintaining hand hygiene. The antimicrobial properties of these products are attributed to the alcohol’s ability to denature proteins. Typically, these products contain a concentration of alcohol ranging from 60% to 95%, along with a thickening agent or humectant such as polyacrylic acid, glycerin, or propylene glycol, which helps reduce the drying effect of the alcohol [25,26,27,28,29,30,31]. These sanitizers have been proven to exhibit microbiological activity against bacteria, fungi, and certain enveloped viruses, including human immunodeficiency virus, herpesvirus, adenovirus, influenza, and parainfluenza viruses [28,30,31]. However, it has been shown that some commercial alcohol-based hand sanitizers contain an alcohol content below the recommended threshold of 60%. Additionally, the presence of ethyl acetate, isobutanol, methanol, and 3-methyl-butanol, which are not recommended alcohols, was also detected. Consequently, consumers were cautioned that within the wide array of hand sanitizer brands available around the world, there exist certain substandard formulations and others that contain traces of toxic substances [32,33]. Another category of immediate hand hygiene products, known as alcohol-free hand sanitizers, has gained increasing attention [34,35]. These products are formulated without alcohol and instead utilize compounds such as povidone-iodine (PVPI), triclosan, or quaternary ammonium. While historically considered to be less effective than alcohol-based counterparts, more recent formulations containing benzalkonium chloride have demonstrated several advantages over alcohol-based products. These advantages include residual antimicrobial activity even after use, a reduced drying effect on the skin of the hands, and consistent efficacy even with repeated use [24].

Organic acids such as acetic acid, citric acid, tartaric acid, lactic acid, and oxalic acid fall under the classification of GRAS (generally recognized as safe) are natural antimicrobial substances that are commonly utilized in the food industry [36]. These organic acids offer certain advantages over sodium hypochlorite and hydrogen peroxide, primarily due to their toxicological safety and environmentally friendly properties [37,38,39]. Among the extensively studied organic acids are acetic acid, lactic acid, citric acid, malic acid, and peracetic acid. These acids are typically employed at concentrations ranging from 0.04% to 2% [40,41]. They have demonstrated bacteriostatic and/or bactericidal effects against significant foodborne pathogens such as *L. monocytogenes*, *E. coli*, and *Salmonella* spp. The primary mechanism of action for these organic acids is the disruption of cell function, achieved by diffusing across bacterial cell membranes and reaching the interior of the cell [42].

Notwithstanding, a solution consisting of a solitary organic acid component is incapable of entirely eradicating or diminishing the virulence of a spectrum of pathogens that have the ability to form enduring biofilms. Consequently, organic acids are typically utilized in conjunction with other antibacterial agents or antibiotics to produce a synergistic impact that can effectively eliminate pathogens [43]. Very recently, it has been shown that the amalgamation of organic acids exerts a synergistic inhibitory influence primarily by inducing multiple destructive effects on the cellular barrier, thereby exhibiting synergistic anti-biofilm effects. Notably, the three-three combination of acetic, lactic acid, and a third organic acid has been found to elicit a superior synergistic antibacterial effect compared to the two-pair combination of acetic and lactic acid [44]. A study was undertaken to assess the efficacy of ethanol-based hand sanitizers, both with and without organic acids, in eliminating detectable rhinovirus from hands and preventing experimental rhinovirus infection [45]. The inclusion of organic acids in the ethanol formulation resulted in a sustained virucidal effect that lasted for a minimum of 4 h [45]. Given the previous demonstration of residual activity against rhinovirus using organic acids [46], it is justifiable to assert that the synergistic amalgamation of organic acids could potentially offer extended residual viral protection in comparison to alcohol-based formulations. However, the efficacy of this residual activity in conferring protection against infection in the natural environment is yet to be ascertained [46,47]. Moreover, numerous pharmaceutical antiseptic formulations incorporate salts as an additive due to their ability to eliminate certain types of bacteria. This is achieved using the salt extracting water from the bacteria, a process called osmosis. As a result, water exits the bacterium in order to equalize the salt concentrations on both sides of its cell membrane. In the absence of water, bacterial proteins, including enzymes, are unable to function properly, leading to the eventual collapse of the cell.

In this study, we have chosen to merge azelaic, citric, and lactic acids with ammonium sulfate salt in order to generate a synergistic antibacterial effect against prevalent foodborne pathogens. The amalgamation of these organic acids with ammonium sulfate in a water medium facilitates the creation of a unique electrolyte or salt/acid solution. Recently, salt/acid solutions have been suggested as alternative methods for preventing the proliferation of microorganisms in food products [48]. Additionally, ammonium sulfate alone demonstrated the ability to impede the growth of mycotoxigenic fungi [48,49,50,51,52]. The hand sanitizer formulation was enhanced using the addition of a small quantity of povidone-iodine, as povidone-iodine (PVPI) has long been recognized for its efficacy as a broad-spectrum microbicide against bacteria, fungi, protozoans, and viruses [53].

## 2. Materials and Methods

### 2.1. Materials

Betadine^®^ Solution (povidone-iodine, 10%), which is a topical aqueous solution of 10% povidone-iodine was purchased from a local pharmacy and used without any modification. American Chemical Society (ACS) reagent, ≥99.5% citric acid, ACS reagent, ≥85% lactic acid, average M_n_ 400 polyethylene glycol (PEG 400), 98% azelaic acid flakes, ACS reagent, ≥99.0% ammonium sulfate crystals were all purchased from Merck, Milan, Italy and used without further modification. In all the formulations, deionized Milli-DI^®^ Water Purification System (9 V battery) water was used.

### 2.2. Sanitizer Solution Formulations

Four distinct aqueous solutions were prepared and labeled as Solution A to Solution D. The composition of all the solutions is summarized in Table 1. Solutions A to C were utilized as control solutions for bacterial time-kill curves, while Solution D served as the hand sanitizer solution. Due to the limited solubility of azelaic acid in water (0.25 g/100 mL [54]), an initial solution consisting of 50 mL of water, 0.5 g azelaic acid, and 2 mL of PEG 400 was prepared. The azelaic acid flakes were dissolved by heating the solution to approximately 70 °C. Subsequently, as the solution gradually cooled to room temperature, citric and lactic acids were added and dispersed according to Table 1. Once the solution reached room temperature, ammonium nitrate crystals were added and rapidly dissolved. Additional water was then added to achieve a final solution volume of 150 mL. At this stage, povidone-iodine was introduced via dropwise addition, and the final solution volume was adjusted to 200 mL. To guarantee the stability of the complexed iodine in the iodophor (PVPI) within the solution, the stability of the solution was assessed by analyzing the UV-vis spectral characteristics of the dispersed PVPI in the solution over a duration of 30 days. The concentration of each component was determined by weighing the ingredients using the precise Advanced MS-TS Analytical Balance from Thermo Fisher Scientific Inc., Italy. The weight percentages were carefully calculated to ensure that the concentration of each organic acid and salt did not surpass the recommended guidelines (not exceeding 1 wt. %) stated in COMMISSION REGULATION (EU) 2020/1419 of 7 October 2020.

### 2.3. Evaluation of Antibacterial Activity Using Agar Well-Diffusion

All the tested strains were bought from Microbiologics, St. Cloud, MN, USA, except for *C. botulinum*, which was provided by Saniter Gida A.S., Turkiye. For *Staphylococcus aureus* (WDCM 00034), *Listeria monocytogenes* (WDCM 00021), and *Escherichia coli* (WDCM 00013), growth conditions of 37 °C for 24 h in Mueller-Hinton agar (MHA) was utilized and the positive control was made with Tetracycline 30 μg/disc. For *Clostridium botulinum* (ISS CNRB CL 14NT), growth conditions of 37 °C for 24–48 h under anaerobic conditions in Mueller-Hinton agar with 5% defibrinated sheep blood (MHAB) was used, and as a positive control, Penicillin G 10 UI/disc was utilized. A suspension of 0.5 McFarland in 0.9% sterile saline solution was prepared for each organism. Subsequently, 100 microliters of the suspension were evenly distributed on each quadrant of MHA and 5% MHAB plates (Oxoid Limited, Basingstoke, UK) using a swab.

Holes with a diameter of 6–8 mm were created in the plates by removing the medium with a sterilized cork borer. Then, 50 microliters of the extract solution in sterile demineralized water (1000 mg/mL) were inoculated into the holes. The plates were incubated under the growth conditions as specified above. A negative control was established using the same suspension used to prepare the extract solution (sterile demineralized water), while antibiotic discs (Oxoid Limited, Basingstoke, UK) were used as positive controls, as detailed at the start of the paragraph. After the designated incubation period, the presence and diameter of the inhibition zone were assessed using a gauge (in millimeters). The measurements were conducted in triplicate to determine the average inhibition zone, and standard deviations were calculated.

### 2.4. Minimal Inhibitory Concentration (MIC) and Minimal Bactericidal Concentration (MBC) Experiments

MICs and MBCs were measured using a standard broth microdilution technique in accordance with the guidelines set forth by the Clinical Laboratory Standards Institute (CLSI) [55]. Bacterial suspensions were prepared by adjusting the number of bacteria to 10^5^ colony-forming units per milliliter (CFU/mL) with fresh Mueller-Hinton broth containing 5% blood (Sheep Blood Defibrinated sourced from Labo Center, Istanbul, Turkiye). Aliquots of each suspension were added to 96-well microplates (Starlab International GmbH, Hamburg, Germany) containing the same volumes of two-fold serial dilutions (ranging from 1.0 to 0.0078 g/mL) of the extracts. Additionally, the controls were established, as shown in Table 1, including the antibiotic-positive controls (with culture medium and bacterial suspension). The plates were incubated for 48 h at 37 °C under anaerobic conditions (Whitley anaerobic jar—3.0 L from Sümer Analitik ve Medikal Teknolojiler San. Tic. A.Ş., Turkiye).

The MIC was defined as the lowest concentration of extract that produced no bacterial growth when compared to time 0 wells. The MBC was determined by sub-culturing the broths used for MIC determination. A quantity of 10 microliters of broth culture from the wells corresponding to the MIC and the higher MIC concentrations was plated onto fresh 5% Sheep Blood agar dishes and then incubated for 48 h at 37 °C under anaerobic conditions. The MBC was represented as the smallest amount of extract that was capable of killing the bacterial inoculum, demonstrated by the total absence of growth [56]. All tests were performed in triplicate, and the results were expressed as means ± standard deviation.

### 2.5. Bacterial Growth Characteristics and Time-Kill Curves

The time-kill test was conducted to characterize the antiseptic efficacy of the hand sanitizer against the chosen foodborne pathogens. The bactericidal effect is defined as a reduction of at least 3-log or the elimination of 99.9% of viable cells within a specific time period [56]. Following the guidelines outlined in the CLSI document M26-A [55], three broth cultures were prepared in Mueller-Hinton broth with 5% blood, each containing a bacterial concentration of 10^5^ CFU/mL. The solutions A to D were added to the first two broth cultures, respectively, to achieve a final concentration of 1 × MIC. The third broth culture served as a control for growth (CTRL). The broth cultures were incubated at 37 °C under anaerobic conditions and at various time intervals (0–2–4–6–8–12–24 and 48 h), the viable cells (CFU/mL) were enumerated. All time-kill curve experiments were conducted in triplicate, and the results were expressed as a Log of viable cell numbers (Log CFU/mL), with standard deviations calculated. The obtained growth curves were analyzed using Growthcurver, an R package for obtaining interpretable metrics from microbial growth curves by fitting the experimental data to the Baranyi and Roberts model [57]. From the resulting curves, the initial values (Log CFU/mL), duration of the lag phase (λ) in hours, maximum growth rate (μmax) (Log CFU/mL/h), and final values (Log CFU/mL) were calculated. In cases where bacteria were not detected at the level of 1.00 Log CFU/mL (<10 CFU/mL), a value of −0.50 Log CFU/mL was assigned [56,57].

### 2.6. S. aureus Biofilm Preparation and Microscopy

Soybean trypsin broth (TSB) with a pH of 7.3 was acquired from Labor Teknik A. S. in Turkiye. In order to assess the efficacy of the hand sanitizer in eradicating biofilms, cultures of *S. aureus* that had been incubated overnight were diluted 1:100 with fresh TSB media. Subsequently, 200 µL of this diluted culture was added to each well of flat-bottom 96-well plates, along with 2% (wt/vol) glucose, which has been reported to promote biofilm formation [58]. The plates were then incubated at 37 ℃ for 24 h to allow for the formation of mature biofilms. After removing the supernatant, 200 µL of TSB broth containing the hand sanitizer was added to each well. Following another 24 h incubation period, the supernatant was removed and the biofilms were quantified using crystal violet staining. This involved adding 200 µL of a 0.25% crystal violet solution to each well and allowing it to stain the biofilms for 15 min. The excess dye solution was then removed, and the biofilms were washed with saline and dissolved in 95% ethanol.

The overnight culture was introduced into a 6-well cell culture plate (Corning Costar, Cambridge, MA, USA) containing serial concentrations of the hand sanitizer solution and incubated statically with 18 mm × 18 mm sterile glass cover slides at 37 °C for 24 h. The biofilms formed on the coverslips were stained with SYTO 9 (green) and PI (red) (Thermo Fisher Scientific, Istanbul, Turkey) at a final concentration of 10 μM. After staining for 15 min in the dark and fixing on object slides, the coverslips were photographed by confocal laser scanning microscopy (Zeiss LSM 980, Jena, Germany). Picture processing and image quantization were completed using ImageJ software.

For the purpose of electron microscopy, the biofilms were delicately excised and placed onto TEM grids. In order to prevent any disruption of the biofilm during the subsequent TEM embedding process, the membranes containing the biofilm were meticulously sliced with a razor blade and then placed onto a drop of 3% sodium alginate (in 0.05 M Hepes buffer, pH 7.4), which was immediately solidified with CaCl_2_ 0.2 M. The samples were then repeatedly rinsed in the same Hepes buffer, postfixed in 1% osmium tetroxide for 1 h at 4 °C, dehydrated in ethanol, and embedded in London White resin (Agar Scientific, Essex, UK). Ultrathin sections were obtained using a Reichert Jung Ultracut E microtome and stained with 3% uranyl-acetate and lead citrate. Observations were conducted using an FEI 120kV HCTEM transmission electron microscope (FEI, Munich GmbH, Germany) operating at 80 kV. For *L. monocytogenes* microscopy, the bacterial culture treated with 1.5 × MIC hand sanitizer solution for 10 h was placed on Formvar-coated copper grids (Agar Scientific Ltd., London, UK) and negatively stained with a 2% aqueous solution of potassium phosphotungstate (Merck, Istanbul, Turkiye).

## 3. Results and Discussion

### 3.1. Stability Testing of the Solutions

The long-term stability of alcohol-free hand sanitizer should be investigated in order to ensure that the initial efficacy levels are maintained and that the ability to kill bacteria remains effective even after repeated use and storage for extended periods of time. According to Kobayashi et al. [59], manufacturers typically assign an expiration date to these hand sanitizers. However, in most cases, the expiration date after opening has not been specified due to variations in frequency of use and environmental conditions, such as temperature and humidity, at different locations. Consequently, each product tends to establish its own expiration date after opening, such as “3 months after opening” or “6 months after opening”. Nevertheless, these expiration dates are determined arbitrarily, and there is currently a lack of scientific evidence regarding the actual concentration of active ingredients and the disinfection efficacy after opening. In this study, we monitored the changes in the pH, UV-vis spectra, MIC, MBC, and spectrophotometric decrease in absorbance at 600 nm corresponding to the transition from a phase-bright spore to a phase-dark spore [60], particularly for challenging-to-eliminate *C. botulinum* spores, for up to 30 days. The absence of alcohol in the current formulations can be considered beneficial, as all of the antiseptics used in Table 1 are solids and do not evaporate like alcohol.

**Figure 1:**
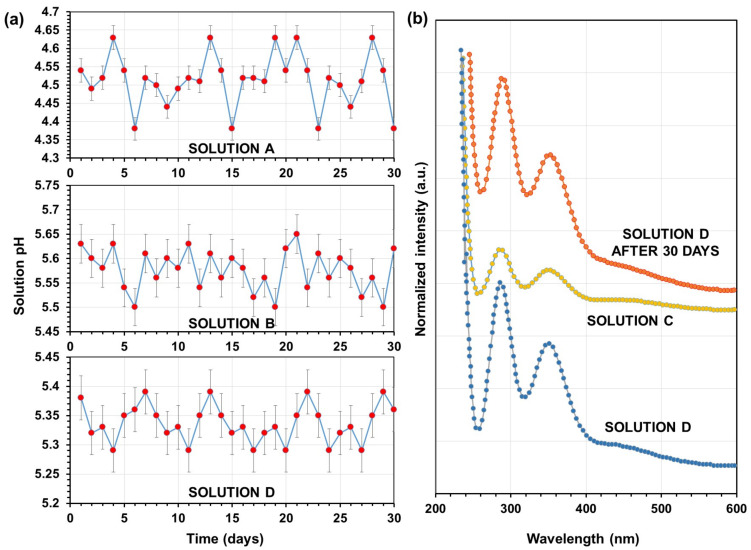
(**a**) pH changes as a result of aging of solutions A, B, and D over a one-month period. (**b**) FTIR spectra of solutions C and D and solution D after one month. Note that Solution D is the hand sanitizer.

The pH monitoring results for solutions A, B, and D over a period of one month are depicted in Figure 1a. Solution A, which contains all the organic acids along with PEG400, exhibits an average pH of approximately 4.52. However, the pH of this solution fluctuated between 4.65 and 4.35. This variability can be attributed to the weak ionization of organic acids when dissolved in water. It is well-known that organic acids solvated in water form hydrogen bonds with long lifetimes as solutes [61]. The fluctuations in pH observed in solution A may be a result of the dynamic establishment or rearrangement of hydrogen bonds between the various organic acids in water [62]. Further investigation is required to fully understand this phenomenon. Nevertheless, as depicted in Figure 1a, no loss of acidity was observed over the course of one month. Solution B does not contain any organic acids. Instead, it consists of PEG400 and ammonium sulfate, which is a salt solution with a slightly acidic nature due to the presence of sulfate ions. Research has shown that the ion-dipole interactions between ammonium sulfate and water are stronger than the hydrogen bonds between alcohol and water [63]. As a result, the pH of this solution is higher, averaging around 5.6, and remains relatively stable. On the other hand, the pH of the hand sanitizer (solution D) fluctuates, as depicted in Figure 1a. The average pH of the solution is approximately 5.35, indicating a slightly acidic nature compared to Solution B. However, it is worth noting that the presence of ammonium sulfate prevents a significant reduction in the pH of the solution due to additional organic acids. This observation can be attributed to the fact that as long as NH_4_^+^ ions are not depleted from the aqueous organic acid-ammonium sulfate solutions, the pH of the medium will not be lowered unless exposed to sudden high heat [64].

The stability of povidone-iodine solutions (PVPI) surpasses that of iodine tincture or Lugol’s solution. It has been observed that aqueous solutions of the complex, which contain 2% available iodine, exhibit no changes when stored at room temperature for a duration of one year [65]. PVPI is known to be more effective in low pH levels [65]. For instance, the optimization of ophthalmic PVPI application can be achieved by adjusting the pH of the formulation to 7.0 using phosphate buffer. This adjustment effectively reduces irritancy while simultaneously ensuring sufficient antibacterial efficacy and storage stability. The antibacterial effectiveness of povidone-iodine was diminished as the pH of the formulation was elevated from 4.0 to 7.0, albeit its overall activity was maintained. Ultimately, povidone-iodine exhibited stability in both normal saline and phosphate buffer for a duration of 30 days [66]. Therefore, it is imperative that the hand sanitizer solution exhibits stability in the presence of organic acids and ammonium sulfate while also demonstrating strong efficacy.

Nevertheless, we conducted a thorough monitoring of the UV-vis spectrum of the hand sanitizer over a period of one month to ensure that the complexed iodine in PVPI did not undergo any undesirable interactions with the other components of the solution. Figure 1b depicts the UV spectra of solution C, which is pure diluted PVPI, and solution D (hand sanitizer) immediately after preparation and after one month of storage under room conditions. The presence of absorption bands at wavelengths 290 and 350 nm is a distinguishing feature of both aqueous solutions of iodine and iodophor solutions like PVPI. In the case of PVPI, a bathochromic shift is present and causes the absorption maximums to move from 290 and 350 nm to 295 and 355 nm, respectively, due to the formation of new complexes between triiodide and the organic acids. This complex exhibits a highly pronounced color, and the intensity of the color in its solutions increases with higher concentrations of iodine [67]. Solution C clearly exhibits these peaks, and notably, the intensity of these peaks is significantly amplified in solution D. This phenomenon can be attributed to the alteration in the solution’s pH caused by organic acids and the presence of ionized species. These factors potentially enhance the rotational and vibrational energy states in the polymer-iodine bonds. Additionally, pH variations can induce changes in the energy difference between the lowest unoccupied molecular orbital (LUMO) and the highest occupied molecular orbital (HOMO), leading to a shift towards longer or shorter wavelengths, accompanied by an augmentation in absorption intensity.

**Figure 2:**
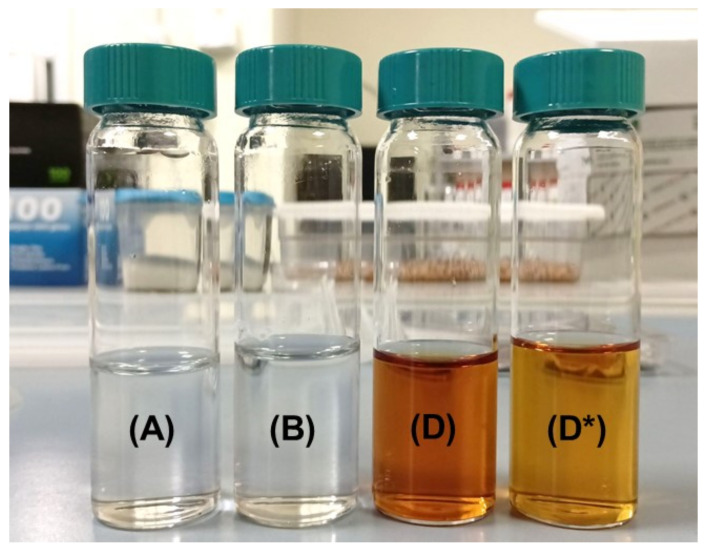
Photographs of solutions A, B, and D in closed glass vials. The label D* shows the solution D after one month of storage with a shift in its color, indicating the formation of triiodide ions in the solution.

The photographs depicting the solutions A, B, D, and D* (after a storage period of 60 days) are presented in Figure 2. As observed in Figure 2, the hand sanitizer exhibits a characteristic light orange color compared to the initial fresh state, which can be attributed to the higher concentration of triiodide (I_3_^−^) ions in the solution. This was also measured as a slight red shift in the absorption peaks after 30 days, and it seems that longer aging results in more triiodide (I_3_^−^) ions in solutions. This state of the solution holds potential advantages, as recent studies have demonstrated that antimicrobial agents containing triiodide complexes with halogen bonding have the ability to release free iodine molecules in a controlled manner. This controlled release occurs via interactions with the plasma membrane of microorganisms, resulting in alterations to the structure of the triiodide anion [68].

### 3.2. Bacterial Growth Inhibition

**Figure 3:**
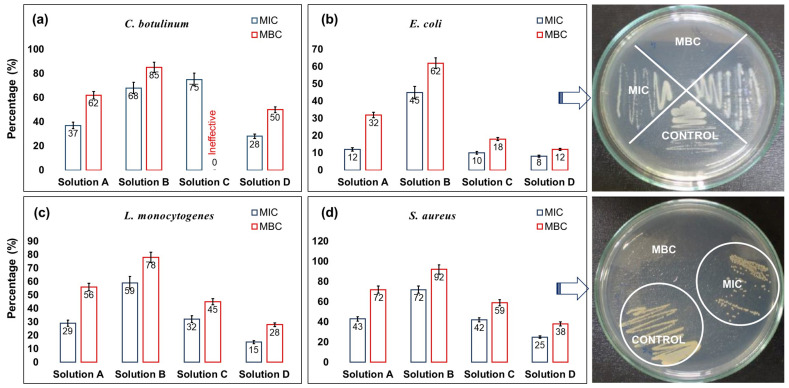
Frequency distribution of MIC and MBC for all the pathogens studied. (**a**) *C. botulinum*, (**b**) *E. coli*, (**c**) *L. monocytogenes*, (**d**) *S. aureus*. The photographs next to the plots (**b**,**d**) on the right show the representative disk diffusion assay results from solution A against *E. coli* (**b**) and *S. aureus* (**d**).

The minimum inhibitory concentration (MIC) is a widely accepted criterion for assessing the susceptibility of organisms to inhibitors. Several factors, such as temperature, inoculum size, and organism type, can influence the obtained MIC value. The minimum bactericidal concentration (MBC) is a complementary test to the MIC. While the MIC test determines the lowest concentration of antimicrobial agent that significantly inhibits growth, the MBC test determines the lowest concentration of antimicrobial agent that results in microbial death [69]. Figure 3 displays the MIC and MBC values as a percentage of the solution required to achieve the desired effect for each solution tested on all four pathogens. It is evident that solution A, which consists of a combination of organic acids, only exhibits significant MIC and MBC values on all pathogens individually, with the highest effectiveness observed against *E. coli* and *L. monocytogenes*.

The successful utilization of organic acids as antimicrobial agents in food materials relies on various characteristic properties of the acids, including their chemical formula, physical form, pKa value, molecular weight, minimum inhibitory concentration, the nature of the microorganism, buffering properties of the food, and the duration of acid-food exposure. Since the non-dissociated portion of the acid is responsible for its antimicrobial effect, the pKa dissociation constant plays a crucial role. The pKa values of many acids typically fall within the pH range of 3 to 5, making it more advantageous to employ acidulants within this range. Additionally, it has been demonstrated that acids with fewer than seven carbons exhibit greater efficacy at lower pH levels, while those with more than eight carbons are more effective at neutral or alkaline pH values. Previous studies have reported that citric acid possesses stronger inhibitory properties on a molar basis compared to lactic and acetic acid [70,71].

Azelaic acid has been found to possess significant anti-inflammatory and antioxidative effects, and it exhibits bactericidal activity against a wide range of Gram-negative and Gram-positive microorganisms, including antibiotic-resistant strains [72]. Furthermore, it is worth noting that ammonium sulfate salt has been found to exhibit mild antifungal properties [51].

The combination of organic acids with this mild antifungal salt has proven to be effective against all the pathogens examined in this study, as shown in Figure 3. Interestingly, solution B also demonstrated efficacy against the pathogens but with higher MIC and MBC dilution percentages (weaker efficacy compared to solution A). The interaction between PEG400 and ammonium sulfate can occur via the formation of hydrogen bonds between the ammonia ions and the hydroxyl groups in PEG400. This interaction can result in the formation of an ionic polymer-like system and may even lead to phase separation in water at certain concentrations [73]. Poly-ionic liquids derived from PEG400 have been shown to possess antibacterial and anti-biofouling properties [74]. On its own, the diluted PVPI solution (solution C) did not exhibit effectiveness against *C. botulinum*, but it did display MIC and MBC values against the other pathogens. However, when combined with solution A as a hand sanitizer, it demonstrated the lowest percentage of MIC and MBC levels for all the tested pathogens. Therefore, it can be concluded that the combination of organic acids, PVPI, and ammonium sulfate can yield highly effective alcohol-free aqueous hygienic solutions. The photographs depicted in Figure 3b,d illustrate the stages of Minimum Inhibitory Concentration (MIC) and Minimum Bactericidal Concentration (MBC) for *E. coli* and *S. aureus* following treatment with solution A.

### 3.3. Long-Term Bacterial Growth Inhibition

**Figure 4:**
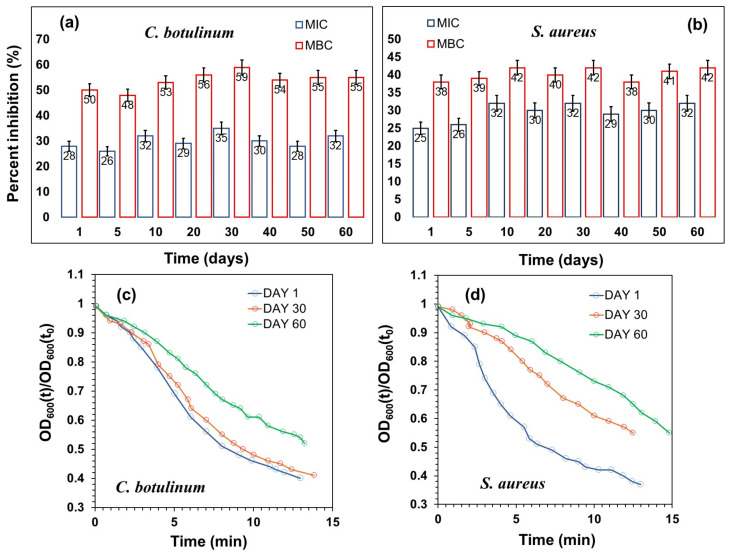
Aging performance of the hand sanitizer over a period of 60 days. MIC and MBC levels were measured at different times for *C. botulinum* (**a**) and *S. aureus* (**b**) pathogens. Optical density plots showing the initial kinetics of the decline phase in *C. botulinum* (**c**) and *S. aureus* (**d**) pathogens measured at day 1, day 30, and day 60 treated with the 1.5 × MIC hand sanitizer.

Subsequently, the antibacterial efficacy of the hand sanitizer was assessed in terms of percentage changes in MIC and MBC values against *C. botulinum* and *S. aureus* (Figure 4a,b) over a period of 60 days while stored under room conditions in light-blocking plastic bottles. It is evident that there was minimal alteration observed in the MIC and MBC performance in both cases. Statistically speaking, a slight increase in the percentage values was inevitable.

However, the results indicate that the hand sanitizer solution remains stable in terms of efficacy performance. Additionally, it is both interesting and informative to examine the initial antibacterial effect kinetics (early stages) of the fresh and aged hand sanitizer solution against the same two pathogens. This can be conducted by measuring the optical density changes in the bacterial cultures treated with antiseptics at concentrations beyond the MIC levels after the “stationary phase”, also known as the death phase. Figure 4c,d displays the kinetics of *C. botulinum* spore termination and *S. aureus* cell proliferation conducted at a 1.5 × MIC level (lower than MBC values). The bacterial proliferation kinetics of *S. aureus* are more pronounced within the first 15 min of treatment with the hand sanitizer. As the solution ages, for example, after one month, the rate of its initial antibacterial effect becomes slower, although it continues to function effectively after 15 min. In the case of *C. botulinum*, a one-month-aged solution performs as effectively as the fresh solution against the spores in terms of rate, and after 60 days, the rate of its bactericidal effect diminishes. It is encouraging to note that during the preliminary kinetics measurements, no lag phase or reversal in proliferation rates was observed under conditions of 1.5 × MIC. Note that the OD_600_(t)/OD_600_(t_0_) values remained constant at about 1.0 at any other MIC level less than 1.5.

### 3.4. Biofouling Tests

**Figure 5:**
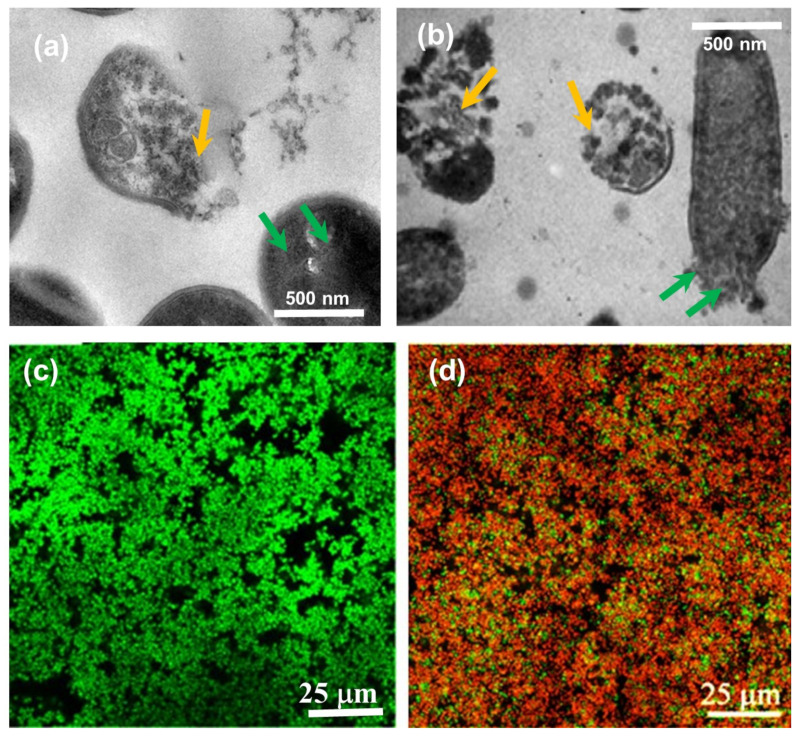
(**a**) TEM image showing the membrane disruption of hand sanitizer treated (10 h) *S. aureus* cells. Mesosome-like structures (green arrows), cell membrane damage, and cell lysis (yellow arrows) are notable. (**b**) TEM image showing the cytological effects of 1.5 × MIC hand sanitizer treated (after 10 h) on *L. monocytogenes*. (**c**) Healthy untreated *S. aureus* cell colonies were photographed using confocal microscopy and dye staining as control biofilm. (**d**) Upon treatment with the 1.5 × MIC hand sanitizer solution after 10 h even though biofilm densities increased, many cells were dead, indicated by the propidium iodide red fluorescence.

Next, we conducted an investigation into the efficacy of the hand sanitizer in eradicating *L. monocytogenes* colony growth and, more importantly, in mitigating biofouling caused by *S. aureus* biofilms. The attachment of *S. aureus* to medical implants and host tissue, as well as the establishment of a mature biofilm, are critical factors in the persistence of chronic infections. The formation of a biofilm, which involves the encasement of cells in a polymer-based matrix, reduces susceptibility to antimicrobials and immune defenses, rendering these infections challenging to eliminate. During infection, the dispersal of cells from the biofilm can lead to the spread of the infection to secondary sites and exacerbation of the condition [75,76]. To visually observe the membrane disruption caused by the hand sanitizer, we employed TEM for further analysis. The TEM image depicted in Figure 5a illustrates partial shrinkage, detachment, and collapse of *S. aureus* strains extracted from the hand sanitizer-treated biofilms, with visible leakage of intracellular mass. Furthermore, the TEM image reveals a series of pathological changes in cells, including the formation of mesosome-like structures (indicated by yellow arrows in Figure 5a), cell membrane damage, plasmolysis, disruption of cytoplasm, and cell lysis (indicated by green arrows in Figure 5a) in the presence of 1.5 × MIC hand sanitizer. In parallel, *L. monocytogenes* colonies treated with the hand sanitizer solution exhibited pore formation, cellular disintegration, and extensive damage to the cell envelope, including the membrane and cell wall (Figure 5b), resulting in the leakage of cytoplasmic materials. Additionally, cytoplasmic materials such as nucleic acids, proteins, and ribosomes in the cytoplasm coagulate and aggregate near the cell membrane.

Upon examination using confocal microscopy, it was observed that *S. aureus* bacteria in the biofilm exhibited an increase in propidium iodide (PI) staining, indicating cell death (refer to Figure 5c,d). Notably, in comparison to the control biofilm (untreated, Figure 5c), *S. aureus* displayed a noticeable increase in the number of cells visible on the disc, although the majority of them were deceased. The heightened aggregation of cells following treatment with the hand sanitizer could potentially be attributed to the presence of ammonium sulfate. Previous studies have demonstrated that ammonium may not be toxic to various bacteria, as most bacteria prefer ammonium as a nitrogen source, and certain species even produce ammonium via processes such as N_2_-fixation in rhizobia and cyanobacteria, as well as amino acid fermentation in proteolytic clostridia. However, depending on the specific conditions of the solution, as exemplified by Muller et al. [77], the presence of free (NH_4_)_2_SO_4_ can hinder growth, not necessarily due to an ammonium-specific effect, but potentially due to increased osmolarity or heightened ionic strength of the medium along with the collective synergistic effect of the organic acids and PVPI.

Ammonium sulfate is recognized for its ability to aggregate bacteria, a phenomenon referred to as “salting out” of bacteria from a medium. The degree and speed of aggregation are directly linked to the hydrophobicity of the bacteria. In a similar vein, novel compounds such as antimicrobial proteins and peptides exhibit agglutinating activity, which involves the ability to clump bacteria following treatment. This characteristic is particularly advantageous as agglutinating agents can be employed to confine bacteria to the site of infection, facilitating their elimination by the immune cells of the host [78,79].

### 3.5. Time-Kill Curves

**Figure 6:**
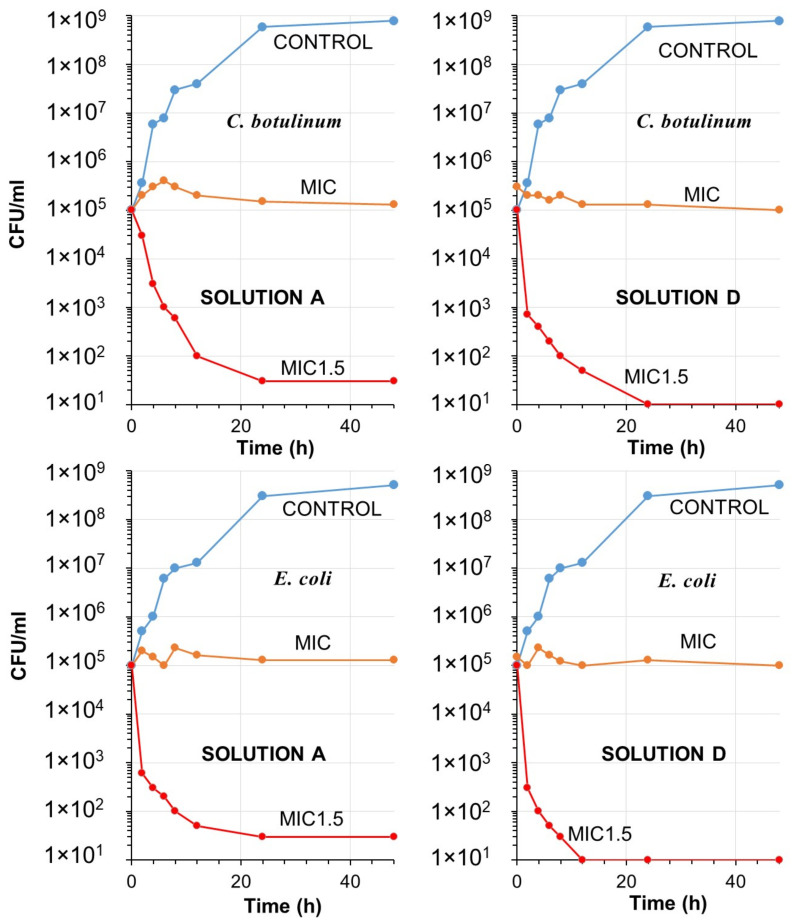
Time-kill curves over a period of 48 h demonstrating the bactericidal activity of *C. botulium* (top panles) and *E. coli* (bottom panels) for Solution A and solution D at MIC, 1.5 × MIC levels, along with non-treated bacterial colonies plotted as control.

**Figure 7:**
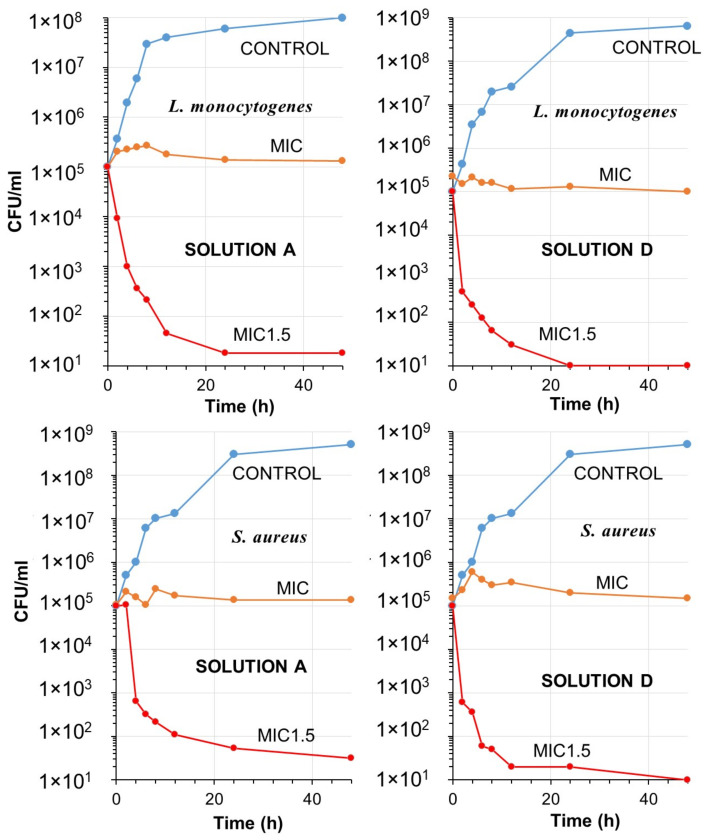
Time-kill curves over a period of 48 h demonstrating the bactericidal activity of *L. monocytogenes* (top panels) and *S. aureus* (bottom panels) for Solution A and solution D at MIC, 1.5 × MIC levels along with the non-treated bacterial colonies plotted as control.

Figure 6 and Figure 7 depict the antibacterial efficacy of solutions A and D against the four specified pathogens at MIC and 1.5 × MIC concentrations. In the case of *C. botulinum*, Solution A, comprising organic acids, PEG400, and salt, exhibited a significant decrease of approximately 3-log in the number of CFU/mL at 1.5 × MIC after 8 h. However, this reduction was not as pronounced after 12 h and did not fully reach a 4-log level (99.99%) even after 48 h (Figure 6). Conversely, the hand sanitizer achieved a 3-log reduction in CFU/mL between 4 to 6 h, with killing levels reaching a 4-log reduction after 24 h. This outcome confirms the enhanced effectiveness of the formulation due to the addition of PVPI. Similarly, for *E. coli*, solution A demonstrated a faster 3-log reduction after 4 h, but did not achieve a complete 4-log reduction thereafter. The hand sanitizer proved highly effective against *E. coli*, as a 4-log reduction could be easily measured after 6 h of contact. Notably, at MIC levels, no killing effect was observed for both pathogens, although no colony increases were detected (Figure 6).

Figure 7 displays the time-kill curves for *L. monocytogenes* and *S. aureus*. Solution A exhibited effectiveness against E. monocytogenes, resulting in a 3-log reduction in less than 6 h. However, it was unable to achieve a 4-log reduction. Conversely, solution D was able to achieve a 4-log reduction after 24 h at the same 1.5 × MIC levels. In the case of *S. aureus*, solution A did not reduce the killing rate to 4-log levels. However, after 48 h of contact, the hand sanitizer was able to achieve a 4-log reduction. Notably, the 4-log reduction in *S. aureus* occurred at a much slower rate compared to other pathogens. Recent studies have indicated that PVPI, when used in conjunction with other antiseptics, may serve as a valuable preoperative decolonizing agent for preventing *S. aureus* infections, including MRSA and mupirocin-resistant strains [80,81].

It should be noted that the time-kill curves of solution A in both figures demonstrate a tailing phenomenon (decline with a lower slope). Solution D contains povidone-iodine, along with the organic acids and salt. The inclusion of this iodophor appears to prevent tailing in the time-kill curves. It can be hypothesized that organic acids are larger in size compared to elemental iodine, allowing iodine to easily penetrate cell walls and lipid barriers in comparison to organic molecules and salt. In the absence of iodine, it is possible that bacteria or pathogens may form clumps or aggregates, hindering the penetration of molecules [82,83]. Consequently, cell subpopulations may arise, capable of tolerating larger molecules that are lethal to individual cells or most of the cells in the initial populations [84].

## 4. Conclusions

A non-alcoholic aqueous solution of hand sanitizer has been developed using a combination of three organic acids—citric, lactic, and azelaic acids. The organic acid blend exhibited a synergistic antibacterial effect against four specific pathogens. However, the efficacy and kill-time curve of the hand sanitizer was enhanced by incorporating ammonium sulfate and povidone-iodine into the formulation. The stability of the solutions was maintained even after being stored in the dark and aged for 60 days. This was attributed to the presence of organic acids and ammonium salt, which resulted in the detection of more triiodide ions in the solutions using visual and spectroscopic analysis upon aging. The presence of triiodide ions is advantageous as they can easily penetrate cell walls and transform into molecular iodine, which is a highly effective antimicrobial agent. Further research is required to determine the optimal MIC and MBC concentrations for a variety of pathogens, and dermatological tests must be conducted to ensure that the formulation is skin-friendly. However, it is safe to say that the sanitizer formulation employs FDA-approved ingredients and can be utilized as a spray or washing agent for fresh produce, meat, and poultry products to safeguard against foodborne pathogens. Finally, we can indicate that although there is a considerable range of variability in material costs when considering a bulk purchase scenario, we can establish certain approximations based on a 70% ethanol solution as a benchmark (unit cost of 1). In this context, the cost of PVPI powder is approximately 7–9 times the unit cost, while azelaic acid is roughly 10–12 times the unit cost. Additionally, citric and lactic acids are approximately 2–3 times the unit cost, whereas ammonium sulfate amounts to half the unit cost. It is important to note that Solution D has all these materials as total solids. Assuming that the total solute concentration in Solution D is approximately 9% and the average cost of the solute or the solids is five units, then the overall cost would amount to approximately 0.45 units.

## Figures and Tables

**Table 1:** Components of the antiseptic aqueous solutions are listed. The proposed hand sanitizer solution is Solution D in the table.

		Quantities in Grams Based on 200.0 g Final Solution Weight			
Ingredients	Function	Solution A	Solution B	Solution C	Solution D
Azelaic acid	Microbicide	0.5	0.0	0.0	0.5
Citric acid	Microbicide	1.0	0.0	0.0	1.0
Lactic acid	Microbicide	1.0	0.0	0.0	2.0
PEG 400	Lubricant	2.0	2.0	0.0	2.0
(NH_4_)_2_SO_4_	Fungicidal	0.0	1.0	0.0	1.0
PVPI solution	Broad spectrum microbicide	0.0	0.0	2.0	2.0
Water	Solvent	195.5	197.0	198.0	191.5

## Data Availability

Data available upon request from the corresponding author.
